# Synthesis of Coral-Like Tantalum Oxide Films via Anodization in Mixed Organic-Inorganic Electrolytes

**DOI:** 10.1371/journal.pone.0066447

**Published:** 2013-06-14

**Authors:** Hongbin Yu, Suiyi Zhu, Xia Yang, Xinhong Wang, Hongwei Sun, Mingxin Huo

**Affiliations:** 1 School of Environment, Northeast Normal University, Changchun, China; 2 College of Resources and Environment, Jilin Agricultural University, Changchun, China; Massey University, New Zealand

## Abstract

We report a simple method to fabricate nano-porous tantalum oxide films via anodization with Ta foils as the anode at room temperature. A mixture of ethylene glycol, phosphoric acid, NH_4_F and H_2_O was used as the electrolyte where the nano-porous tantalum oxide could be synthesized by anodizing a tantalum foil for 1 h at 20 V in a two–electrode configuration. The as-prepared porous film exhibited a continuous, uniform and coral-like morphology. The diameters of pores ranged from 30 nm to 50 nm. The pores interlaced each other and the depth was about 150 nm. After calcination, the as-synthesized amorphous tantalum oxide could be crystallized to the orthorhombic crystal system. As observed in photocatalytic experiments, the coral-like tantalum oxide exhibited a higher photocatalytic activity for the degradation of phenol than that with a compact surface morphology, and the elimination rate of phenol increased by 66.7%.

## Introduction

As a multifunctional material, tantalum oxide has attracted increasing attention in recent years and has been widely used in the fabrication of memory devices [1], capacitors [2], orthopedic instruments [3], and photocatalysts [4], etc. Tantalum oxide deserves such special recognition in many areas mainly because it has so many excellent properties such as wide band gap, high photocatalytic activity under UV irradiation, chemical resistance, high melting point, good mechanical strength, biocompatibility, and so on [3], [4]. Considering the promising application potential of tantalum oxide in industries, great efforts have been motivated to further refine and develop the synthesis techniques of tantalum oxide.

As we know, the preparation of nano scale materials has become a hot research topic owning to the fact that the nanophase material usually possesses some special properties. For example, TaON with the meso–microporous structure could significantly increase the photocatalytic activity [5]. Of late, various nano materials with different morphologies were successfully fabricated, e.g. nanotube [6]–[11], nano pyramidal arrays [12], nano dimple arrays [13], nano hollow spheres [14], [15] and nano coral-like materials [16]–[18]. Many methods could be used to synthesize them. As for the preparation of nano tantalum oxide, the anodization is a very popular and efficient approach that has been widely investigated under different conditions [6]–[11], [19]–[22]. The tantalum oxide films with compact structure could be synthesized in different fluoride–free electrolytes such as mineral acids, organic acids, salt solutions and oxidizing agents at anodization voltages typically between 100 V and 200 V over a wide range of temperatures, which were well documented in the literature [23]. Normally, the introduction of fluoride ion was believed to be essential to the formation of nano morphologies [8]. The structures of nano pores and nanotube could be achieved when the anodization process was performed in the highly corrosive electrolyte such as the mixture of HF and H_2_SO_4_ [6]–[11], [21]–[22]. However, only in the concentrated H_2_SO_4_ was it possible to obtain the nanotubular morphology [6]–[11]. Moreover, it was reported that relatively low concentrations of organic additives would be of benefit to the growth of nanotube [9]. Additionally, high aspect ratio nanoporous Ta_2_O_5_ films could be also prepared in non-aqueous electrolytes consisting of glycerol and NH_4_F [19]. Furthermore, it was reported that the role of water could be described as a “passivator” and small additions of water would result in a compact layer [19], [20]. As mentioned above, the tantalum oxide films with nano-structure were usually synthesized in either highly corrosive electrolytes or non-aqueous organic electrolytes (in which the content of water should be controlled as low as possible). Therefore, to develop some new methods that had advantages of simple operation, mild reaction conditions and easy control would help to boost the application of tantalum oxide.

In this work, we reported for the first time the fabrication of uniform porous films with the coral-like morphology in a mixed organic-inorganic electrolyte (that was the mixture of ethylene glycol, phosphate, NH_4_F and H_2_O). The microstructure and characteristics of the prepared tantalum oxide were investigated by FESEM, XRD and UV–vis absorption spectra. In addition, phenol, a common organic pollutant widely used in pharmaceutical and chemical industries, was chosen as a model pollutant to examine the photocatalytic performance of coral-like tantalum oxide films.

## Materials and Methods

### Materials

Tantalum foils (0.5 mm thickness, 2.5×5.0 cm, purity >99.95%) was purchase from Baoji Xinyao Metal Material Co., Ltd. A platinum foil (0.1 mm thickness, 2.5×5.0 cm, purity >99.95%) purchased from ChangShu ChangHong Precious Metal Co., Ltd. All other reagents purchased from Beijing Chemical Works, such as ethylene glycol (EG), anhydrous ethanol, H_3_PO_4_ and NH_4_F, were of analytical reagent grade and used without further purification. Twice–distilled water was used throughout the experiments.

### Preparation of the Coral-like Tantalum Oxide

The method for preparing the coral-like tantalum oxide (named CLTO) was that a tantalum foil was polished firstly with abrasive papers and ultrasonically degreased in anhydrous ethanol and distilled water for 15 minutes in turn, and dried in nitrogen flow. This pretreated tantalum foil was then anodized for 1 h at 20 V in a two–electrode configuration equipped with a platinum cathode at room temperature. The electrolyte was a mixture of EG, phosphoric acid and NH_4_F (EG +10%H_2_O +0.25%H_3_PO_4_+3%NH_4_F). The obtained CLTO was annealed in air at 550°C for 3 h with a heating and a cooling rate of 1°C min^–1^ to crystallize Ta_2_O_5_.

For the purpose of comparison, the tantalum oxide film with a compact surface morphology (named CSTO) was also fabricated according to the same processes mentioned above except for changing the electrolyte to EG +70%H_2_O +0.25%H_3_PO_4_+3.6%NH_4_F.

### Characterization

The surface morphologies of tantalum oxide films were characterized by a Field Emission Scanning Electron Microscope (FESEM, JSM 7600–F, JEOL, Japan). The solid phases were identified by powder X–ray diffractometry (XRD) with Cu Kα radiation (D/maxZ200PC, Rigaku, Japan). The absorbance spectra were recorded on a UV–Vis diffuse reflectance spectrophotometer (DRS, Cary 50, Varian, America).

### Photocatalytic Experiments

The photocatalytic degradation of phenol by using CLTO and CSTO films as photocatalysts was carried out in a rectangular quartz reactor (40×50×60 mm) with magnetic stirring under UV light irradiation. A 450W high-pressure mercury lamp served as the UV light source. The effective area of photocatalyst was 10 cm^2^. At a given time interval, 1.0 mL aliquots were sampled to examine the residual phenol by a HPLC (Agilent 1200) equipped with a C_18_ column. Methanol and water (v: v = 80∶ 20) served as the mobile phase and the flow rate was 1.0 mL min^−1^. The wavelength of detection was 270 nm. A total organic carbon (TOC) analyzer (TOC−VCPN, Shimadzu, Japan) was employed for mineralization degree analysis.

## Results and Discussion

### Morphologies and Structural Properties

In general, the tantalum oxide films with nano-structure could be synthesized in non-aqueous organic electrolytes but the content of water in electrolytes needed to be controlled as low as possible [19], [20]. This was because, as reported in the literatures [19], [20], that water in electrolytes could be described as a “passivator”, and small additions of water would result in the formation of compact layers. However, the situation was somewhat different in our studies. That is, the existence of a small amount of water in organic electrolytes was allowed, and nanoporous tantalum oxide films had been synthesized successfully in a mixed organic-inorganic electrolyte.

**Figure 1:**
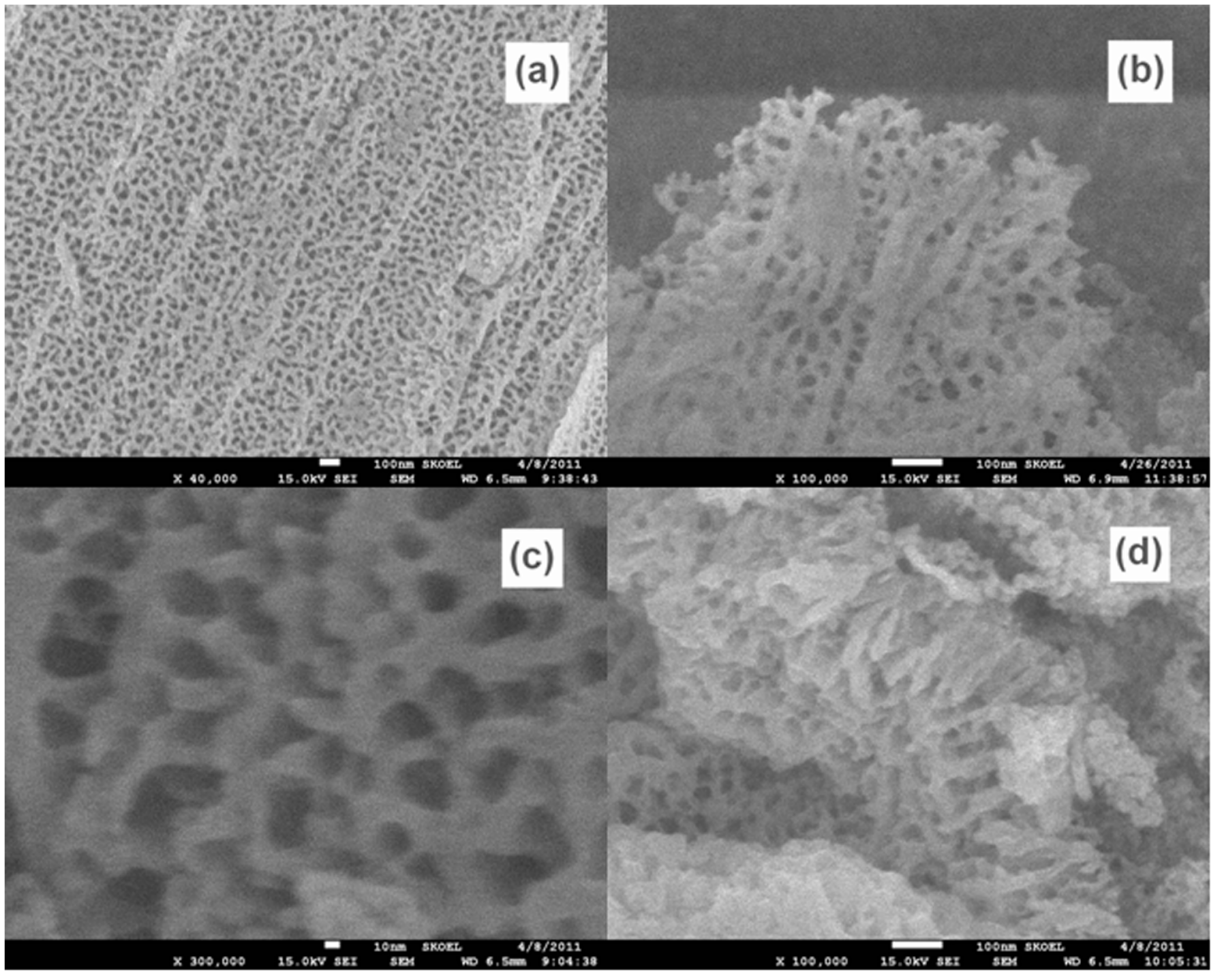
SEM images of the CLTO films formed in the electrolyte of EG +10%H_2_O +0.25%H_3_PO_4_+3%NH_4_F. (a) top view; (b) a fragment (c) the magnification of (a); (d) cross-sectional view.

Fig. 1 shows the morphologies of the tantalum oxide films fabricated in the electrolyte of EG +10%H_2_O +0.25%H_3_PO_4_+3%NH_4_F. From the SEM image of Fig. 1(a), it can be seen that the tantalum oxide film appears to be uniform and many nano-scaled pores distribute continuously throughout the film, exhibiting a coral-like morphology (named CLTO). Fig. 1(b) gives the SEM image of a CLTO fragment and it looks more like a fan coral. The diameter of pores ranges from 30 nm to 50 nm. The magnification of the CLTO is shown in Fig. 1(c) where we can get more details. As displayed in Fig. 1(c), the surface of CLTO films is not very smooth but there are many nanorods stretch out the plane where nanopores locate. This can be seen more clearly from the cross-sectional view (Fig. 1(d)). In fact, these nanopores or channels interlaced each other and the depth was about 150 nm. As compared with the images in Fig. 1(a) and (b), the tantalum oxide in Fig. 1(c) and (d) looks more like horny coral. The coral-like films, with a nanoscaled rough surface, will provide more surface sites for photocatalysis and enhance the photocatalytic ability.

**Figure 2:**
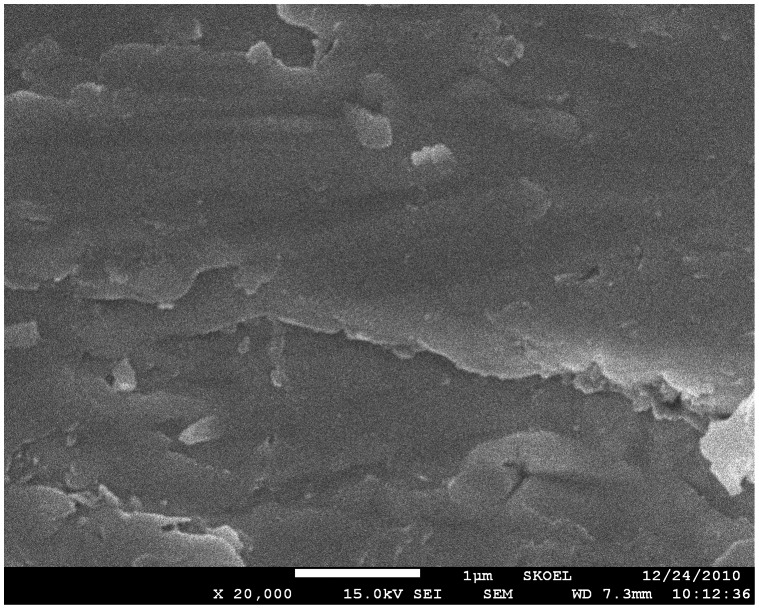
SEM image of the CSTO film formed in the electrolyte of EG +70%H_2_O +0.25%H_3_PO_4_+3.6%NH_4_F.

We had also discovered in the experiments that compact tantalum oxide films would be formed for higher contents of water. As shown in Fig. 2, a compact film was obtained when the electrolyte containing 70% H_2_O was used. In this case, large quantity of water suppressed the growth of nanopores.

**Figure 3:**
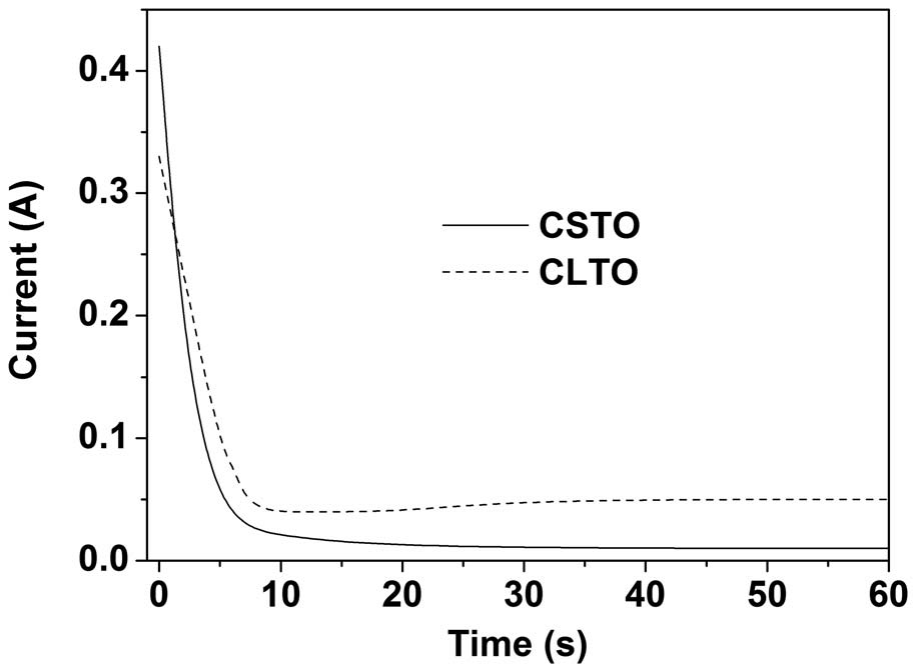
Current variation obtained during the preparation of CLTO and CSTO. The total anodization time was 1 h.

Fig. 3 gives the current variation obtained during the preparation of CLTO and CSTO. In both cases, the current decreased sharply at first, which was similar to what observed in the anodization of valve metals in literatures [8], [9], [11], [23], [24]. This was mainly because of the formation of a compact oxide barrier layer with poor electrical conductivity, as shown in reaction 1 for Ta [8], [23].
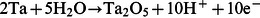
(1)

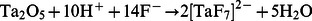
(2)


After that, the current leveled off gradually, but the current obtained in the preparation of CLTO was slightly higher than that recorded in the synthesis of CSTO. This result, to some extent, could reflect the difference of structure between these two films. In the fluoride-containing electrolyte, fluoride ions could chemically attack tantalum oxide (reaction 2), leading to the increase in current with time [8], [9], [23]. Currently, it is widely believed that the growth of porous oxide films resulted from the competition between the oxide growth and its dissolution. As far as chemical reactions (reaction 1 and 2) are concerned, the addition of water would promote the formation of Ta_2_O_5_ and, conversely, suppress its dissolution. This might be one of the reasons why the water usually served as a “passivator” especially in non-aqueous electrolytes [19], [20].

In our experiments, the addition of large amount of water resulted in the appearance of a compact film (CSTO). This is because the tantalum oxide in the solution with high proportion of water had insufficient dissolution rate and thus did not dissolve concurrently with the creation of compact oxide films although the amount of fluoride used was a little more than that in the anodization of CLTO. More importantly, according to the “field focusing effect” and the phenomenon of “local, self-induced acidification” [23], high viscosity solutions help to maintain the dissolution of oxide films at certain sites, thus facilitating the formation of porous oxide films (including nanotube structure). In present work, compared with the preparation of CSTO films, low proportion of water undoubtedly increased the viscosity of electrolytes during the synthesis of CLTO films, and thus the porous morphology appeared.

**Figure 4:**
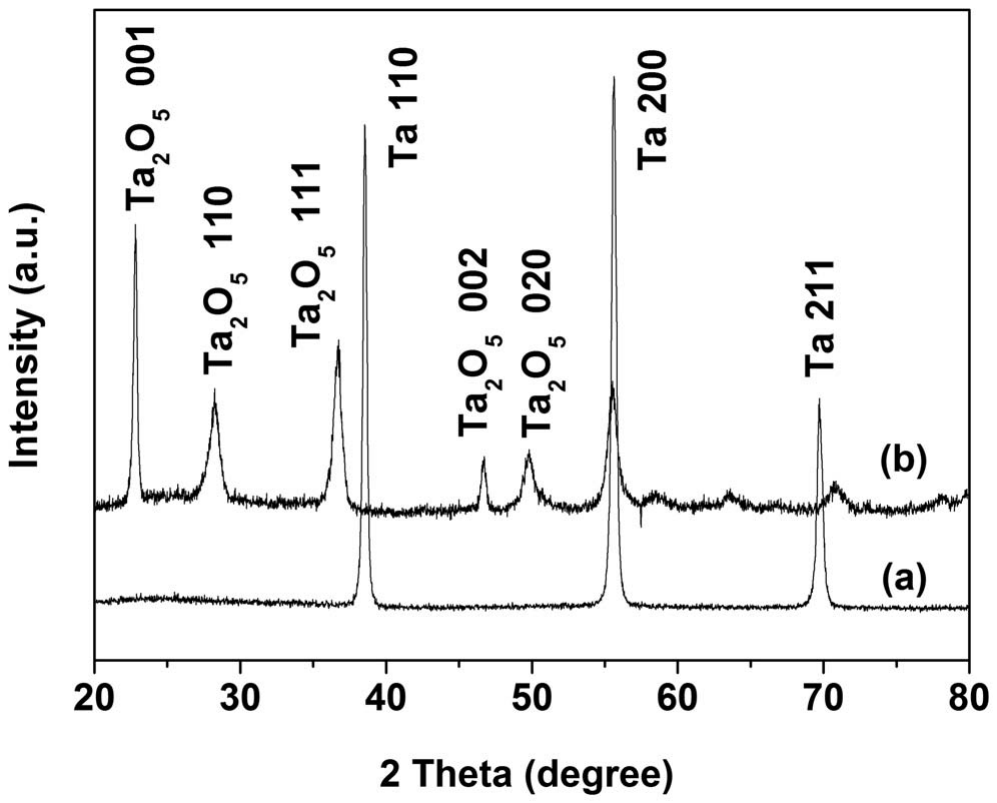
XRD patterns of (a) the as-prepared coral-like Ta_2_O_5_ and (b) the calcined sample.

XRD analysis is a useful technique that can be used to identify the crystalline structure of samples. As displayed in Fig. 4, the as-prepared CLTO film was amorphous due to that no discernible diffraction peaks were observed in its XRD pattern besides the diffraction peaks of Ta substrate (JCPDS, No. 89–5158). However, for the sample obtained after calcinations, the appearance of some new diffraction peaks was indicative of the formation of Ta_2_O_5_ with orthorhombic phase according to the standard diffraction data (JCPDS, No. 89–2843). Based on the Scherrer’s equation and the full width half-maximum peak of (001) crystal plane, the average crystallite size was estimated at around 27.6 nm.

**Figure 5:**
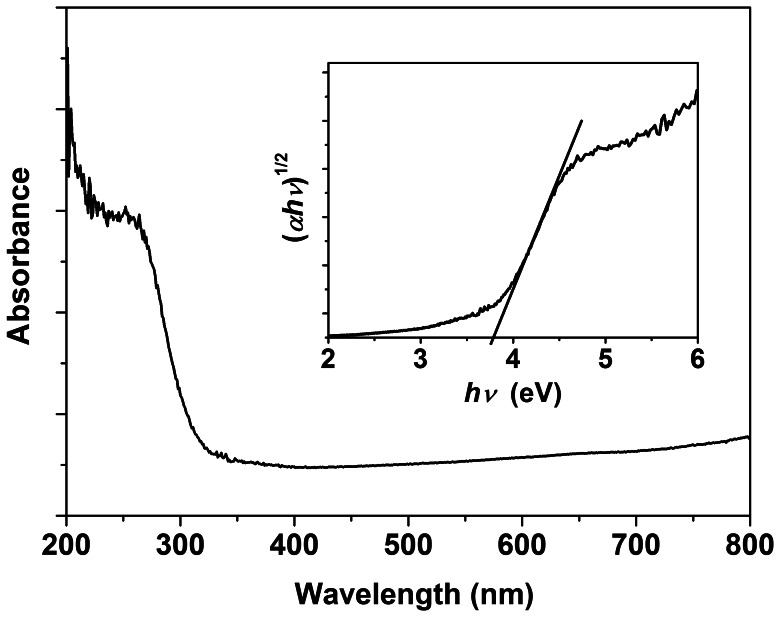
DRS spectrum of the annealed coral-like Ta_2_O_5_. The insert is the corresponding variation of (*αhv*)^1/2^ vs. *hv*.

In order to determine photo-absorbance properties, the annealed CLTO film was analyzed by DRS in the wavelength range of 200–800 nm. As shown in Fig. 5, the absorption spectrum demonstrated a higher absorbance in the near UV region, and the absorption band edge was around 320 nm. The band gap (*E*_g_) of an indirect gap semiconductor (Ta_2_O_5_ for instance) can be determined by the equation [25]:
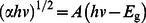
(3)where *hv* is the photon energy, *α* is the absorption coefficient, *A* is a constant relative to the material. The corresponding (*αhv*)^1/2^∼*hv* curve was illustrated in Fig. 5 (insert), and the band gap of CLTO was around 3.8 eV which was similar to the value reported in the literature [14].

### Photocatalytic Experiments

**Figure 6:**
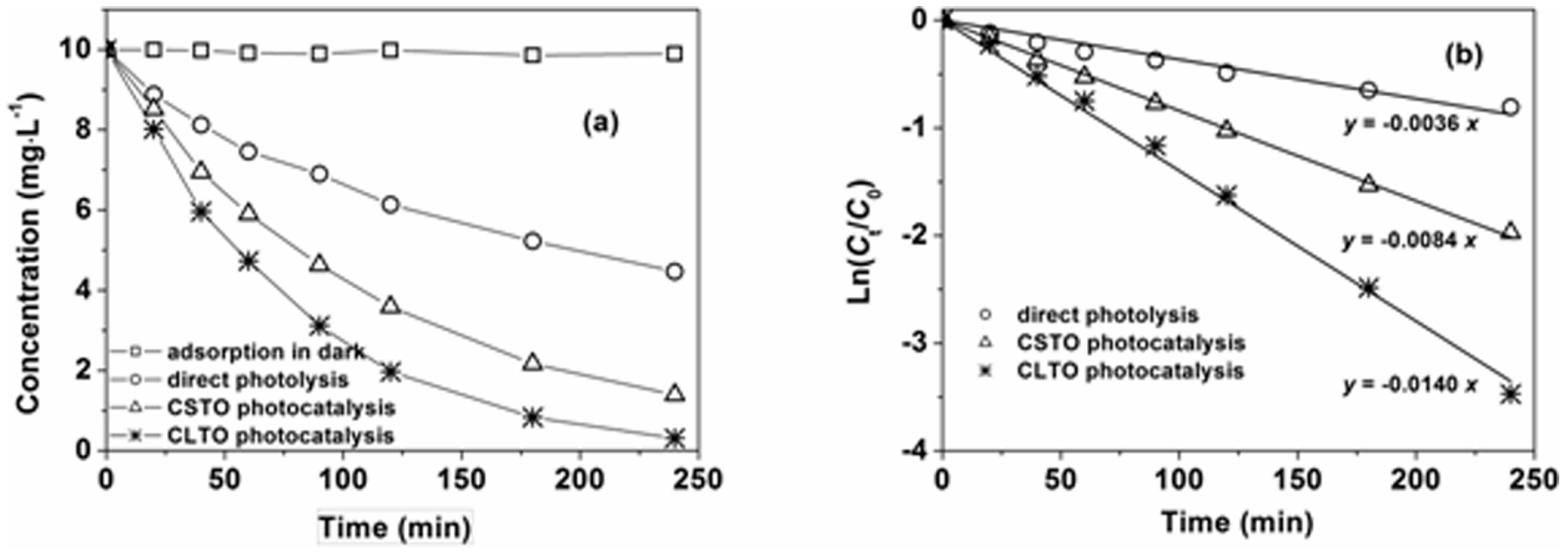
Variation of (a) phenol and (b) ln(*C*_0_/*C*_t_) vs. reaction time with or without photocatalysts.

The photocatalytic activity of the CLTO films was evaluated by degrading phenol with CSTO films as controlled photocatalysts. Firstly, the results obtained in dark conditions indicated that the adsorption of phenol could be negligible. Additionally, phenol was able to be decomposed through direct photolysis, as seen from Fig. 6(a), but the elimination rate was relatively slow (55.3% in 4 h). As compared with direct photolysis, phenol dissipated more rapidly when photocatalysts were adopted. For the CSTO films, 86.1% of phenol was photocatalytically degraded. However, a higher percent of phenol (96.9%) could be removed when the CLTO films were used in the photocatalysis. It could be seen from Fig. 6(b) that the elimination of phenol followed pseudo first–order kinetics. Namely, ln(*C*_0_/*C*_t_) was linear with the reaction time. The kinetic constant for CLTO photocatalysis was around 1.67 and 3.89 times that for the CSTO photocatalysis and the direct photolysis, respectively. TOC is a measure of organically bounded carbon, and the parameter of TOC removal is usually selected to evaluate photocatalytic abilities of catalysts. In present work, 43.3% of TOC was mineralized after 4 h irradiation with CLTO films as photocatalysts, whereas only 28.4% of TOC was transformed into CO_2_ in the case of CSTO films. The results obtained here demonstrated that the photocatalyst with nano porous structure evidently enhanced the photocatalytic abilities. The main reason for this improvement was that, as compared with the two-dimensional plane of CSTO films, the CLTO films with three-dimensional microstructures could provide higher surface areas and more active sites on which more organic matters could be catalytically degraded.

### Conclusions

A nano porous tantalum oxide film with coral-like morphology was successfully fabricated by simply anodizing a tantalum foil in a mixed organic-inorganic electrolyte containing ethylene glycol, H_3_PO_4_, NH_4_F and H_2_O. Pore diameters in the range of 30 to 50 nm were observed. The pores interlaced each other and the depth was about 150 nm. The photocatalytic experiments revealed that, compared with CSTO films, phenol could be more efficiently degraded by using CLTO films as photocatalysts. The enhanced photocatalytic activity was attributed to the fact that three-dimensional microstructures involved in the CLTO films could provide higher surface areas and more active sites.
